# Mixed Organic Solvents Induce Renal Injury in Rats

**DOI:** 10.1371/journal.pone.0045873

**Published:** 2012-09-21

**Authors:** Weisong Qin, Zhongxiu Xu, Yizhou Lu, Caihong Zeng, Chunxia Zheng, Shengyu Wang, Zhihong Liu

**Affiliations:** 1 Research Institute of Nephrology, Jinling Hospital, Nanjing University School of Medicine, Nanjing, China; 2 Department of Nephrology, Central Hospital of Zhabei District, Shanghai, China; The University of Manchester, United Kingdom

## Abstract

To investigate the injury effects of organic solvents on kidney, an animal model of Sprague-Dawley (SD) rats treated with mixed organic solvents via inhalation was generated and characterized. The mixed organic solvents consisted of gasoline, dimethylbenzene and formaldehyde (GDF) in the ratio of 2∶2:1, and were used at 12,000 PPM to treat the rats twice a day, each for 3 hours. Proteinuria appeared in the rats after exposure for 5–6 weeks. The incidences of proteinuria in male and female rats after exposure for 12 weeks were 43.8% (7/16) and 25% (4/16), respectively. Urinary N-Acetyl-β-(D)-Glucosaminidase (NAG) activity was increased significantly after exposure for 4 weeks. Histological examination revealed remarkable injuries in the proximal renal tubules, including tubular epithelial cell detachment, cloud swelling and vacuole formation in the proximal tubular cells, as well as proliferation of parietal epithelium and tubular reflux in glomeruli. Ultrastructural examination found that brush border and cytoplasm of tubular epithelial cell were dropped, that tubular epithelial cells were partially disintegrated, and that the mitochondria of tubular epithelial cells were degenerated and lost. In addition to tubular lesions, glomerular damages were also observed, including segmental foot process fusion and loss of foot process covering on glomerular basement membrane (GBM). Immunofluorescence staining indicated that the expression of nephrin and podocin were both decreased after exposure of GDF. In contrast, increased expression of desmin, a marker of podocyte injury, was found in some areas of a glomerulus. TUNEL staining showed that GDF induced apoptosis in tubular cells and glomerular cells. These studies demonstrate that GDF can induce both severe proximal tubular damage and podocyte injury in rats, and the tubular lesions appear earlier than that of glomeruli.

## Introduction

Industrial solvents constitute an indispensable ingredient of modern living. Patients with organic solvents related nephrosis have been reported [1]–[3]. All patients manifest typical nephrosis with severe edema, massive non-alternative proteinuria, hypoalbuminemia and hypercholesterolemia. Renal histological examinations have shown a decreased podocyte density, segmental proliferation of parietal epithelial cells, reflux of tubular cells and loss of proximal tubular brush border. Although some experimental studies in animals with exposure to different hydrocarbons were reported [4]–[6], the renal injury of solvents has not been well confirmed in animal models. Therefore, this study intends to testify whether organic solvents induce injury on proximal tubular cells and podocytes using rat models.

Organic solvents are carbon-based solvents (i.e., they contain carbon in their molecular structure). Many different classes of chemicals can be used as organic solvents, including aliphatic hydrocarbons, aromatic hydrocarbons, amines, esters, ethers, ketones, and nitrated or chlorinated hydrocarbons. Millions of workers are exposed to organic solvents that are used in such products as paints, varnishes, lacquers, adhesives, glues, degreasing/cleaning agents, and in the production of dyes, polymers, plastics, textiles, printing inks, agricultural products, and pharmaceuticals [7], [8].

The effects of a single solvent on kidney have been reported in rats [9], [10]. However the renal injuries were quite different from clinical observations [11], [12]. In real life people are exposed to multiple chemicals or organic solvents simultaneously rather than a single agent, because most chemicals or organic solvents are the mixtures of several different kinds. Some solvents enhance the metabolism of others, while others inhibit metabolism and thereby increase solvents levels in the blood and reduce elimination time [13], [14].

Gasoline is used in cleaning/degreasing agents and was very frequently used in machinery fitters and assemblers as well as in plumbers. Painters usually used gasoline to wash paint brushes and hands in China. Dimethylbenzene is usually used in paints [15] and formaldehyde is the main injury factor in newly renovated house in China. In this study, a rat model of renal injury induced by mixed solvents of gasoline, dimethylbenzene and formaldehyde (GDF) was generated and characterized.

## Results

### Exposure to GDF Induced Proteinuria in Rats

**Figure 1:**
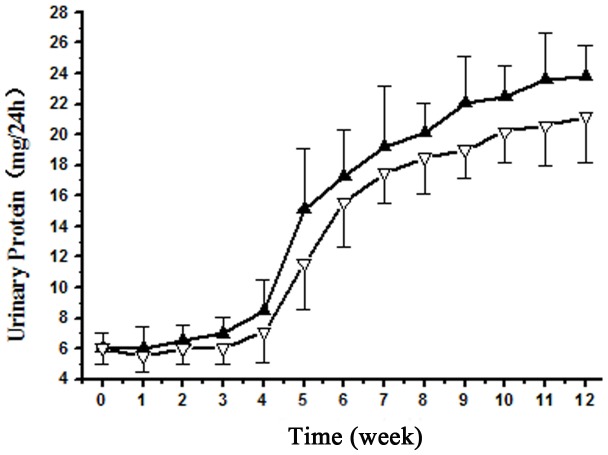
Progression of proteinuria induced by GDF in experimental rats. The urinary protein levels of the proteinuric males (n = 7) and females (n = 4) were shown in solid and empty triangles, respectively.

**Figure 2:**
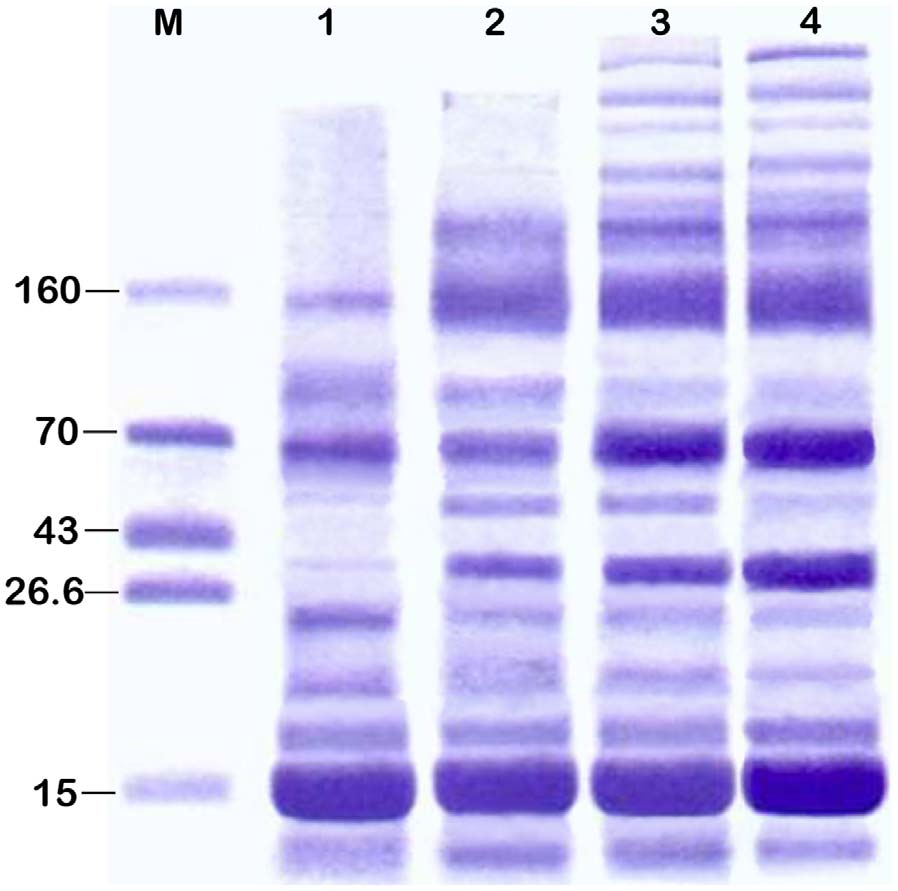
SDS-PAGE analysis of urinary proteins of the rats after exposure to GDF. Lane 1–4 represents urine sample at 8, 10, 11 and 12 weeks, respectively. M. Protein standard (kD).

The urinary protein over 8 mg/24 h in model rats is defined as proteinuria. Proteinuria appeared in rats after exposure to the solvents for 5–6 weeks and increased steadily thereafter (Figure 1). 43.8% of male rats (7/16) and 25% of female rats (4/16) developed proteinuria by 12 weeks of exposure. At this time point, the urinary protein of the proteinuric rats were 23.8±2.4 mg/24 h in males and 21.2±3.1 mg/24 h in females (P<0.05), indicating male rats were more susceptible to the solvents than females in term of proteinuria development. SDS-PAGE analyses of urinary proteins showed that the molecular weights of most proteins were in the range of 6–20 kDa at 8 weeks, and additional bands with MW of over 70 kDa were seen at 12 weeks, suggesting a progressive disruption of filtration barrier (Figure 2, Table 1).

**Table 1:** Size distribution of urinary proteins based on densitometry analysis.

Molecular Weight(kD)	8 w (%) (n = 5)	10 w (%) (n = 5)	11 w (%) (n = 5)	12 w (%) (n = 5)
>70	9.6±5.8	13.5±2.2	15.6±3.6	17.2±5.3
60–70	14.7±9.4	11.7±3.4	16.0±5.4	27.8±13.5
40–60	2.1±2.2	2.9±0.9	2.2±1.7	3.1±2.3
20–40	14.5±5.6	24.1±6.8	19.6±4.9	13.6±2.9
6–20	59.2±10.7	47.9±7.0	46.7±10.2	38.4±10.3*

Note: *P<0.05: Compared with 8 week.

### N-Acetyl-β-(D)-Glucosaminidase (NAG) Activity was Induced in the Urine by GDF

**Figure 3:**
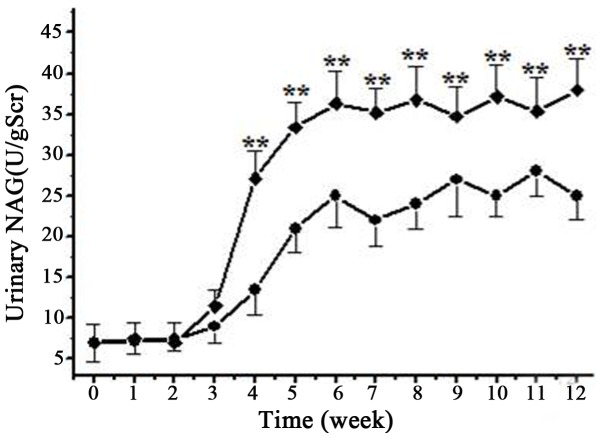
Increase of urinary NAG activity in experimental rats during the treatment with GDF. The urinary NAG activities of male and female rats were shown in squares and circles, respectively. The data are presented as mean±SD. **denotes p<0.01.

To determine if proximal tubules was injured by GDF, we measured the activity of N-Acetyl-β-(D)-Glucosaminidase (NAG), a marker of proximal tubular cell injury, in the urine of the rats. We found NAG activity was significantly increased in the urine after GDF exposure for 4 weeks, and the increase of urinary NAG activity was more prominent in male rats compared with females (Figure 3). At the time point of 6 weeks, NAG activities in male and female rats were 36.3±4.5 U/gScr and 25.2±3.8 U/gScr (P<0.01), respectively. No further increase was observed in the two groups thereafter (Figure 3).

### Renal Histopathological Changes after GDF Exposure

**Figure 4:**
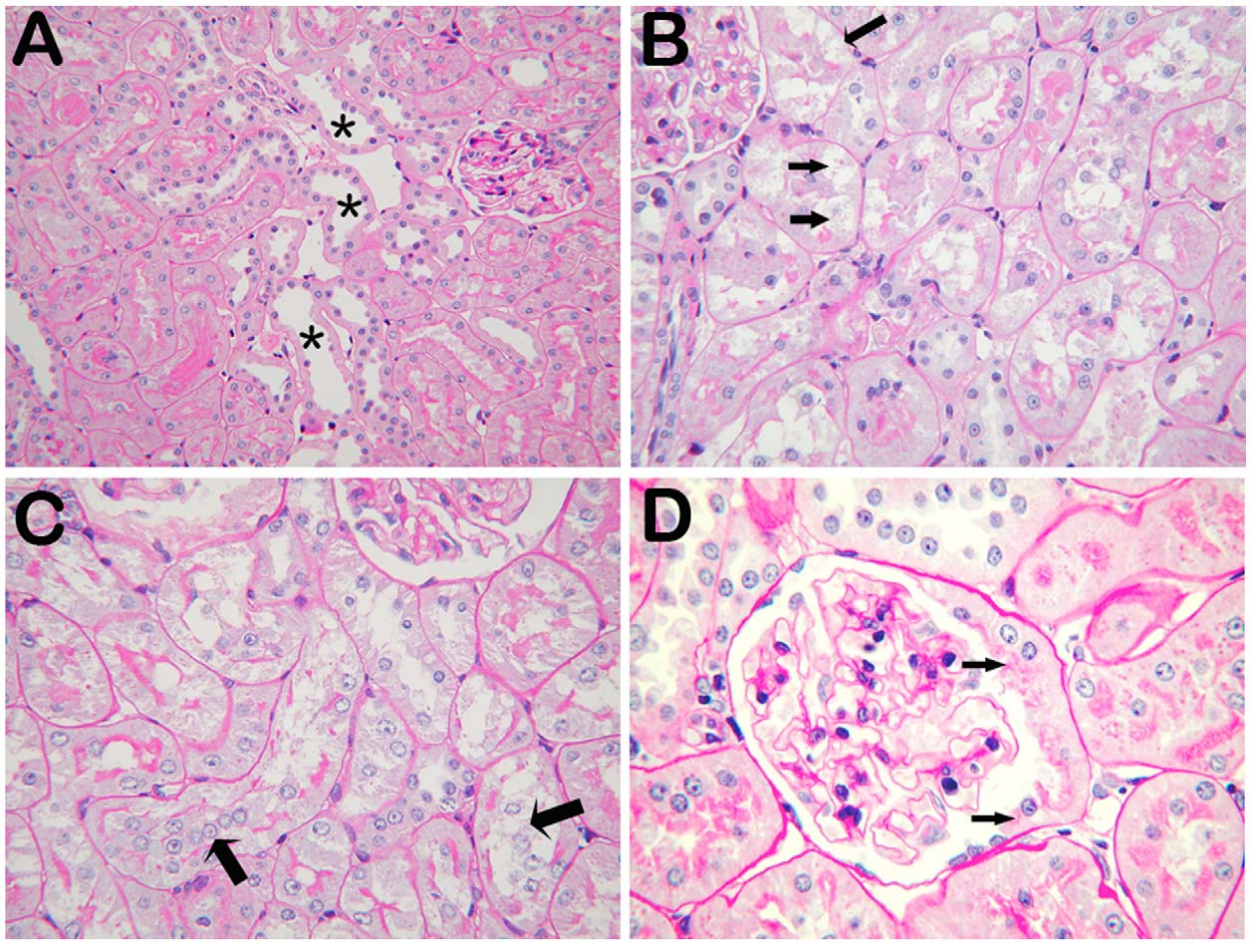
Histological alterations in the kidney of rats with GDF treatment. PAS staining was performed on the renal sections of kidneys. A. Brush border of was lost in some segments of proximal tubules (asterisks). B. Cloud swelling and vacuole formation were also observed in tubular epithelial cells, as indicated by arrows. C. Detached cells, presumably tubular epithelial cells, were found in tubular lumen (arrows). D. Proliferation of parietal epithelium and tubular reflux (arrows) in a glomerulius. (A: 200X; B, C, D: 400X).

After GDF exposure for 6 weeks, the rats were subject to renal histopathological examination. We found a loss of cortical proximal tubular brush border, cloud swelling and vacuole formation in tubular epithelial cells, and proliferation of parietal epithelium and tubular reflux in glomeruli. No overt abnormality was seen in mesangial area (Figure 4).

**Figure 5:**
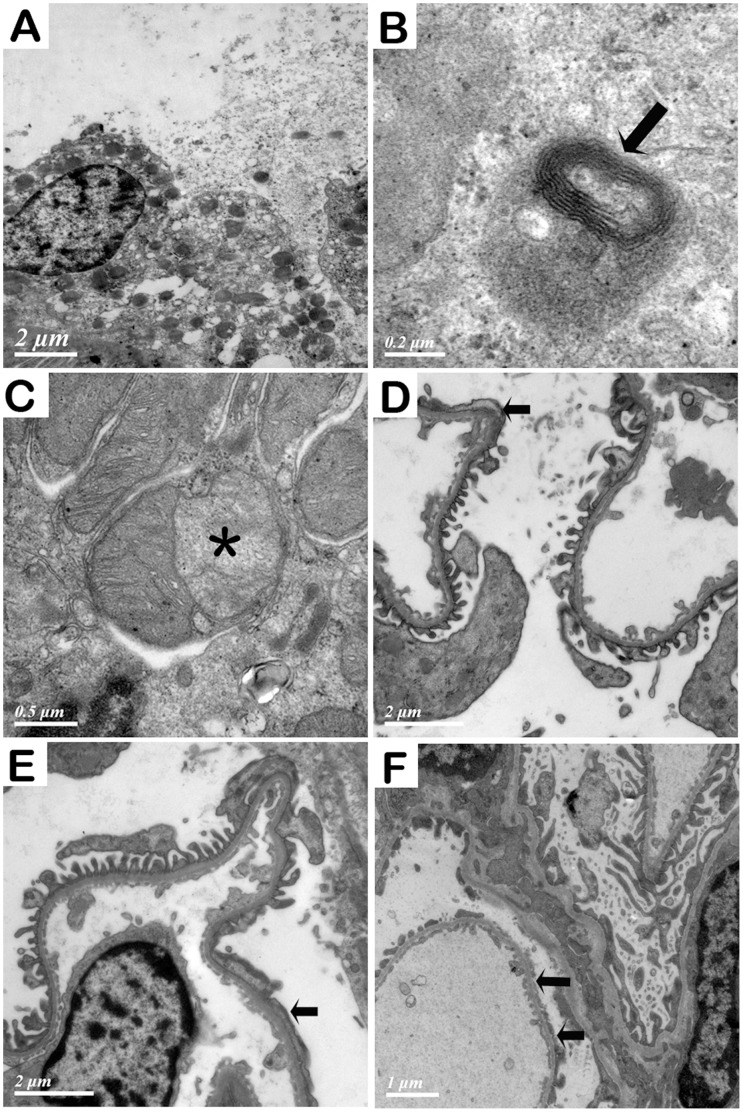
Ultra structural examination of the kidney from the rats with organic solvent treatment. A. Brush border and cytoplasm of tubular epithelial cell were dropped and epithelial cells were disintegrated. B. An autophagolysosome (arrow) in a tubular epithelial cell. C. Mitochondria in a tubular epithelial cell were degenerated. The inner membrane of a mitochondrion was stripped from outer membrane, forming a space in between (asterisk). D, E, F. Foot process of podocytes after exposure to GDF at 8, 10 and 12 weeks, respectively. Segmental foot process fusion at 8 and 10 weeks was indicated with arrows. Partial foot process was stripped from the glomerular basement membrane at 12 weeks (arrows).

Renal ultrastructural examination of the rats with GDF exposure for 6 weeks showed that brush border and cytoplasm of tubular epithelial cells were dropped, resulting in loss of cell integrity, and that tubular epithelial cells were disintegrated. Autophagolysosomes were also found in tubular epithelial cells. Moreover, abnormalities of mitochondria in tubular epithelial cells were obvious, including degeneration, reduction in number, and inner membrane separation from outer membrane. In the glomeruli, we observed segmental foot process fusion of podocytes at 8 and 10 weeks. Partial foot process was stripped from the glomerular base membrane at 12 weeks. In some areas of GBM, foot processes of podocytes were lost, resulting in naked GBM. No electronic dense deposits were seen in mesangial area, subendothelial area or along basement membranes (Figure 5).

### Altered Distribution and Expression of Nephrin and Podocin in GDF Treated Rats

**Figure 6:**
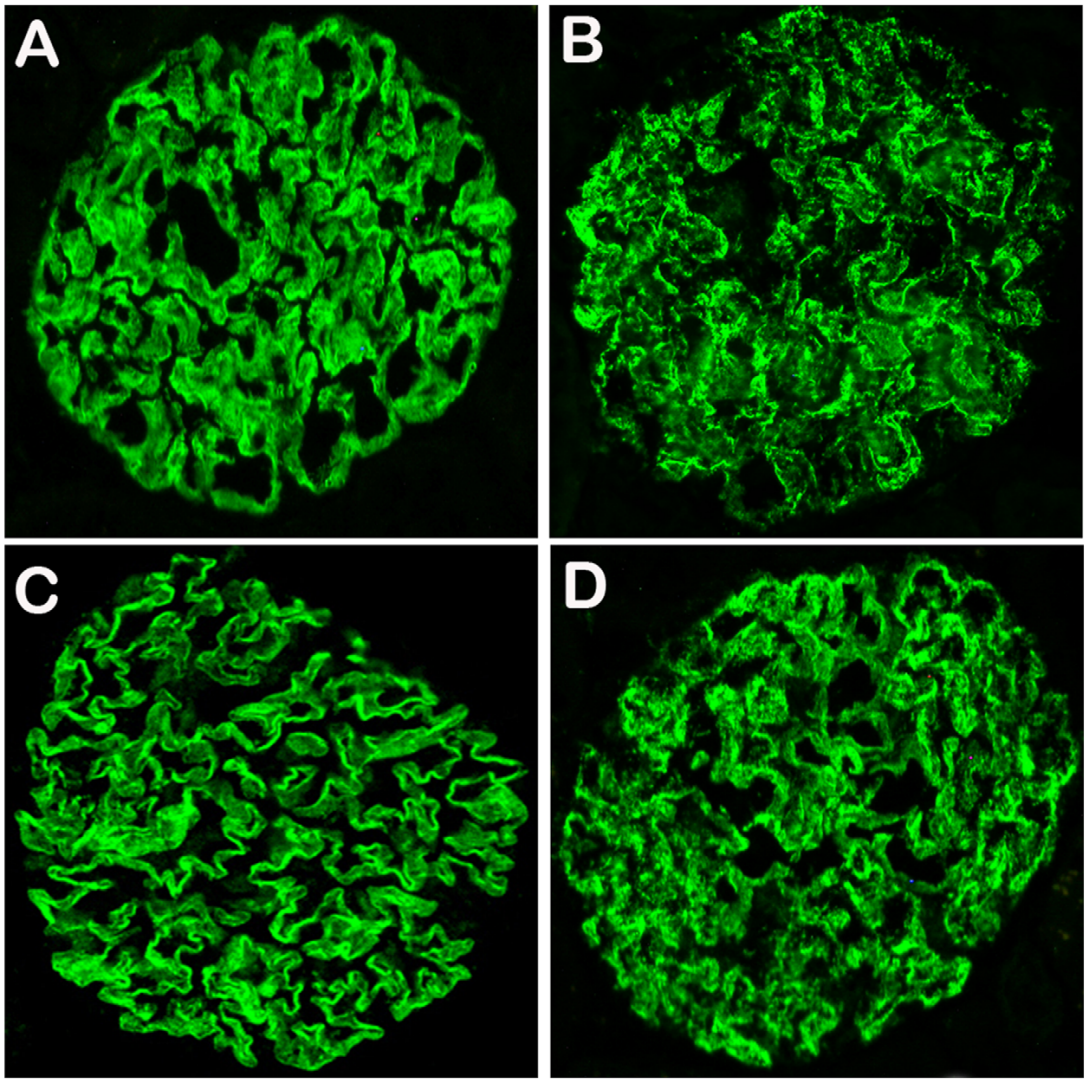
Nephrin and podocin distributions and expressions were changed in the rats after exposure to GDF for 12 weeks. A. Continuous linear pattern of the distribution of nephrin in normal rats. B. Distribution of nephrin was altered in the glomerulus of a rat exposed to GDF, exhibiting an irregular and granular pattern. C. Distribution of podocin in normal rats is similar to nephrin. D. Distribution of podocin in rats exposed to GDF is also similar to nephrin.

Nephrin and podocin are the components of podocyte slit diaphgrams and are sensitive markers for podocyte injury. Therefore, we performed immuno-staining for nephrin and podocin on the kidney of rats with GDF exposure. The distribution of nephrin and podocin on podocytes were continuous and regular along glomerular basement membrane in normal rats. However, in the rats with GDF exposure for 12 weeks distribution of nephrin and podocin was disturbed, as shown by their discontinuous and granular pattern (Figure 6).

### Upregulation of Desmin in the Podocytes of GDF Treated Rats

**Figure 7:**
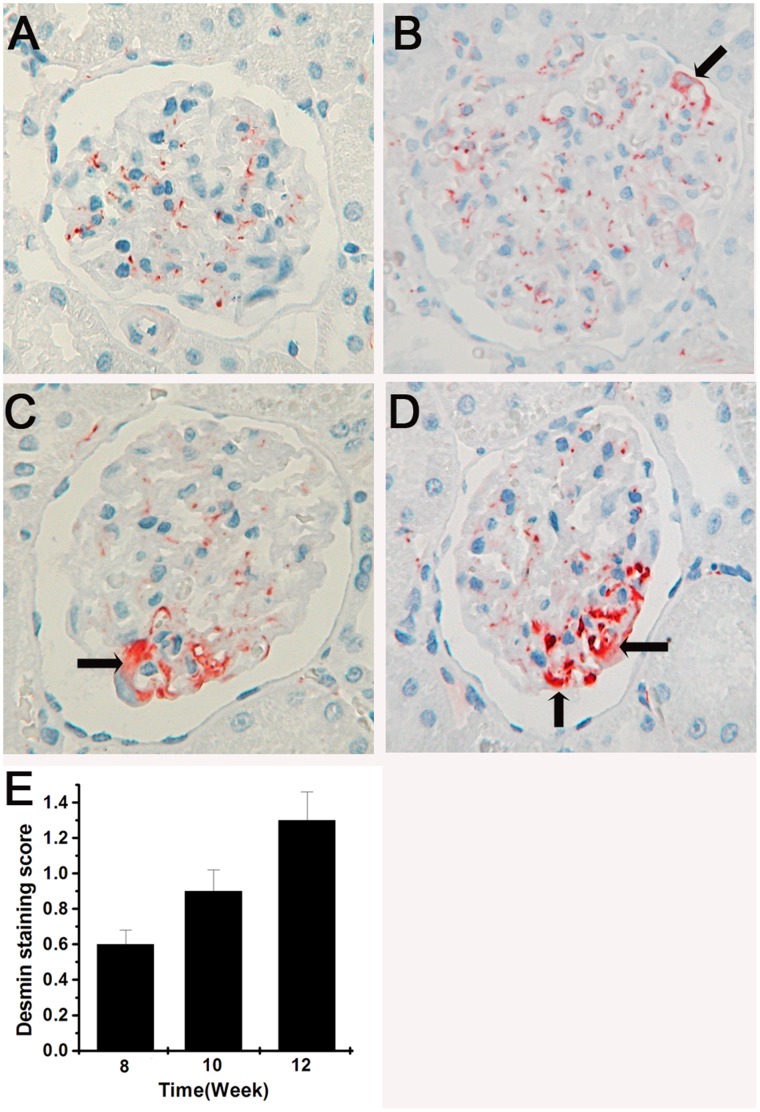
Injury of organic solvents on cytoskeleton. A. No expression of desmin in podocytes of control rats. B, C, and D are representative patterns of desmin expression in experimental rats exposed to GDF for 8, 10 and 12 weeks, respectively. The areas with desmin are pointed by the arrows. E. Semiquantitative analysis of desmin expression at each time point. Mean values±s.e.m. are presented (n = 25 glomeruli per group), P<0.01.

Desmin was used in this study as a marker to indicate cytoskeleton injury of podocytes [16]. In the normal kidney, anti-desmin antibody gave rise to a weak staining in the mesangial cells, but no staining in the podocytes. After exposure to GDF at 8,10 and 12 weeks, a partially enhanced staining of desmin was observed in the podocytes (P<0.01), indicating that a remarkable podocyte cytoskeleton injury was induced by the organic solvents (Figure 7).

### Apoptosis was Developed in the Kidneys of Rats Exposed to GDF

**Figure 8:**
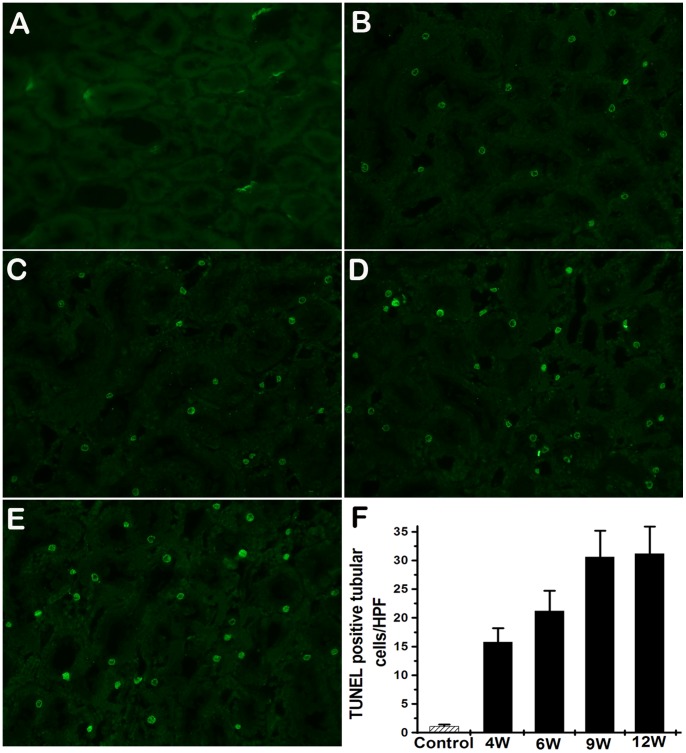
Tubular cells apoptosis in the rats exposed to GDF. A. Kidneys of control rats show no positive nuclear stain for TUNEL. B–E. Kidneys from the rats with GDF treatment show a significant increase in TUNEL-positive staining after exposure to GDF at 4, 6, 9 and 12 weeks, respectively. F. The TUNEL positive staining cells of the kidney tissue were averaged for rats of each group (n = 5). Values represent means ± SD. HPF, high power field.

**Figure 9:**
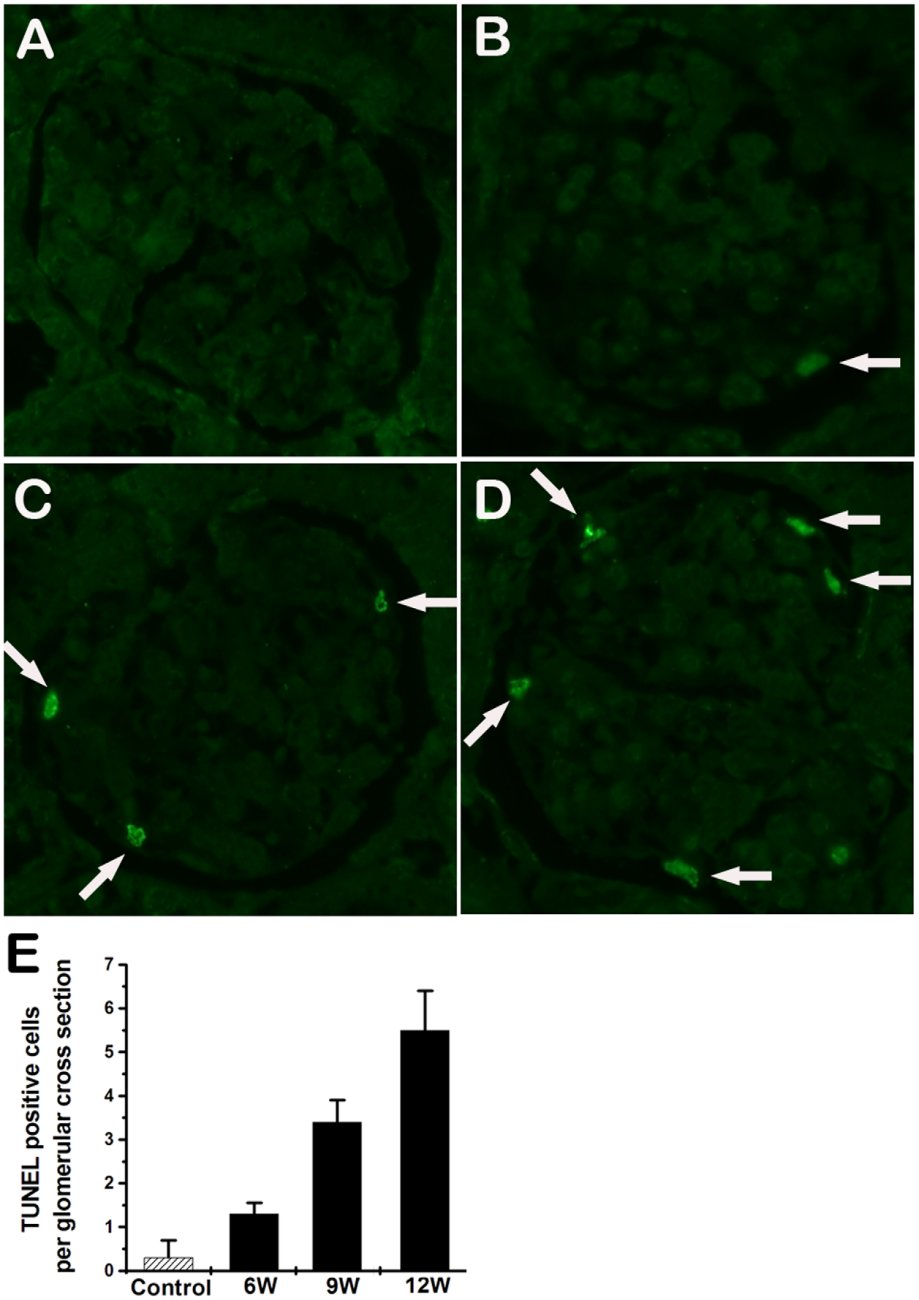
Glomerular cell apoptosis in the rats exposed to GDF. A. Glomeruli from controls show no positive nuclear stain for TUNEL. B-D displays the representative micrographs of TUNEL-stained glomeruli of rats exposed to GDF at 6, 9 and 12 weeks, respectively. Arrows indicate apoptotic cells. E. TUNEL positive staining cells on a glomerular cross section were averaged for rats of each group (n = 5). Values represent means ± SD. (original magnification ×400).

Tubular cell apoptosis, as demonstrated by terminal deoxynucleotidyl transferase dUTP nick end-labeling (TUNEL) staining, was significantly increased since 4 weeks (Figure 8). TUNEL-positive cells were also detected in glomeruli of GDF induced rats (Figure 9). These findings suggest that GDF induced apoptosis in both tubular cells and glomerular cells.

## Discussion

Several studies have examined the role of organic solvent exposure in a population of patients with glomerulonephritis [17]–[22]. Some of these studies have demonstrated a significant association between hydrocarbon exposure and the development of glomerulonephritis, and this association has been further confirmed by more studies. However the causative role of hydrocarbon exposure in the development of glomerulonephritis needs to be verified in animal models. Although organic solvent induced renal injury has been reported, previous studies mainly focused on proximal tubular cells [10], [23]. In this study, organic solvents induce injury on proximal tubuler cells and podocytes in rat models as observed in patients.

In our earlier studies, we used single chemicals, including benzene, toluene, xylene, formaldehyde and trichloromethane, to induce renal injury in rats, and observed only subtle renal injury in the rats with exposure of 4 to 6 weeks, including mild loss of cortical proximal tubular brush border and some detachment of tubular epithelial cells (data not shown).

It is known that the effects of the organic substances contained in a solvent mixture are additive [24], [25]. Even at a low level of exposure to a mixture, the toxicity resulted from the additive effect of several solvents can exceed the toxicity of a single solvent [26]. Since patients are mainly exposed to mixtures composed of many kinds of organic solvents, it is more meaningful to use a mixture of several different kinds of organic solvents for animal model studies. Gasoline is widely used in cleaning equipments. Gasoline is usually used by painters to wash paint brushes and hands in China. Dimethylbenzene is usually contained in paints. Formaldehyde-containing materials are often used in the decoration of houses in China. Therefore, in this study we used the mixture composed of gasoline, dimethylbenzene and formaldehyde (GDF) to induce renal injury in rats. The results of our studies have shown that GDF is able to induce severe renal injuries in rats, including those in both glomeruli and tubules.

The major lesions in the glomeruli of rats exposed to GDF for 12 weeks include proliferation of parietal epithelium and tubular reflux, segmental foot process fusion of podocytes, and decreased expression and altered distribution of nephrin and podocin. GDF also induced injury on cytoskeleton of podocytes, as indicated by the conspicuously enhanced staining for desmin. Consistently, glomerular filtration barrier was found to be disrupted as evidenced by the appearance of proteins with larger molecular weights (over 70 kDa) in urine after exposure to GDF for 12 weeks.

GDF also induced tubular injury in this model. Urinary NAG was increased significantly after GDF exposure for 3–4 weeks, indicating the presence of tubular injury. The tubular injury included loss of cortical proximal tubular brush border, disintegration of tubular epithelial cells, cloud swelling and vacuole formation in tubular cells. In addition, cellular organelles were also injured, including lysosomes as evidenced by the formation of autophagolysosomes in the cells, and mitochondria which degenerated with the inner membrane stripped from outer membrane.

Some mechanisms behind hydrocarbon-induced glomerulonephritis have been proposed [27], [28]. Chemical damage to either the pulmonary capillary alveolar basement membrane or the glomerular basement membrane (GBM) could induce an antigen-antibody response [29]. By combining with renal proteins hydrocarbons may act as haptens and induce autoimmunity against kidney cells [30]. However, these mechanisms were not supported by our study because no electronic dense deposits were observed in mesangial area, subendothial area or along basement membranes.

Organic solvents could induce apoptosis. Toluene or xylene could induce apoptosis of porcine proximal tubular cells [23]. Formaldehyde could induce apoptosis in peripheral blood lymphocytes of personnel working in pathology departments [31]. In this study, tubular cell apoptosis was significantly increased after the exposure to GDF for 4 weeks. TUNEL-positive cells were detected in glomeruli of rats exposed to GDF at 6 weeks. Our results demonstrated that GDF induced apoptosis in both tubular cells and glomerular cells.

The tubular lesions induced by GDF could be observed at 4 weeks of the exposure. The tubular injury appeared much earlier than that of glomeruli, which is due to the unique physiological features of the kidney. Efficient transport systems may lead to an accumulation of chemicals in the renal tissues, especially in the tubular epithelium. The countercurrent flow within the descending and ascending limb increases, or multiplies the osmotic gradient between tubular fluid and interstitial space. For substances with glomerular filtration or/and tubular secretion which are not or poorly resorbed in the tubules, their concentration in the tubular system compared to serum will increase by several times [32]. GDF inhalation initially resulted in the injuries of tubular cells in the kidney. Tubular reflux in glomeruli was observed in this study, which allowed the concentrated solvents in the tubular system to further injure the podocytes. The podocyte damage induced by GDF could be a consequence of tubular injury.

In this study, only 43.8% of male rats and 25% of female rats developed proteinuria after 12 weeks of exposure to GDF. Genetic variations might contribute to the differential susceptibilities of these rats to organic solvent at the same dose [33]. The conversion of solvents to reactive intermediates is mainly based on oxidation by cytochrome P450 family [34], [35]. Genetic polymorphism has been shown for these enzymes. Inherited differences in metabolic capacity play an important role in individual responses to organic solvents [36], [37].

In the present study, male rats developed more severe proteinuria than female rats with the same solvent exposure; in addition, the progressive increase of urinary NAG activity was much more prominent in male rats than in females. These results are consistent with clinical observations that a genetic or sexual predisposition is present in organic solvent induced renal injury. However the underlying mechanisms remain to be elucidated.

In conclusion, this study confirmed that organic solvents could induce apoptosis in both tubular cells and glomerular cells, resulted in both severe proximal tubular damage and podocyte injury in rat models. The tubular lesions induced by organic solvents happened earlier than that of glomeruli.

## Materials and Methods

### Animals

#### Ethics statement

The Institutional Animal Care and Use Committee at Jinling Hospital specifically approved this study and the use of rats.

Adult Sprague-Dawley (SD) rats with body weights of 150 to 180 grams were provided by Experimental Animal Center, Jinling Hospital, Nanjing. Rats were housed under standard conditions including pathogen-free environment and free access to food and water. 24-hour urine samples were collected with metabolic cages in which only water but not food was provided. Gasoline, dimethylbenzene and formaldehyde were mixed in the ratio of 2∶2:1. The mixture (GDF) was placed in an inhalation chamber under dynamic airflow condition, and its concentration was adjusted to 12,000 PPM. Rats were treated with GDF twice a day, each for 3 hours. Rats were sacrificed after ketamine narcosis. 5 male and 5 female rats in model group and control group were killed for histology at 8,10 and 12 weeks, respectively. Renal tissues were processed using the standard method for histological and immunofluorescence microscopy studies.

### SDS-PAGE Analysis of Urine Proteins

Urine proteins were measured by Bradford method and fractionated by SDS-PAGE using 10% polyacrylamide gels. SDS-PAGE was performed with a Mini Protean II Cell system (Bio-Rad, CA, USA). Gels were stained using the Coomassie method and were analyzed in a gel scanner densitometer (Ultroscan XL, Pharmacia, USA).

### Measurement of Urinary NAG

Urinary NAG was measured by colorimetric assay using a commercially available kit (Roche Applied Science) according to the manufacturer’s protocol.

### Histological Studies of Renal Specimens

Kidney tissues were fixed in 10% formalin, dehydrated in graded alcohol and embedded in paraffin. 2 µm sections were cut and stained with hematoxylin and eosin, periodic acid-Schiff regent, periodic acid-sliver methenamine and Masson’s trichrome. All slides were evaluated by the same pathologist who was blinded to the identities of the specimens using a Nikon E800 microscope.

### Electron Microscopy Examination of the Kidney

Blocks of renal cortex tissue (l mm^3^) were fixed in cold 3.75% glutaraldehyde for 4 h. After washed in 0.1 M phosphate buffer (pH 7.5) for 5–6 times, renal tissues were postfixed in 2% osmium tetroxide for 2 h, dehydrated in graded acetone and ethanol, and embedded in epoxy resin (SPI Inc, US). Ultra thin sections (80–90 nm) were stained with uranyl acetate and lead citrate, then examined and photographed in a Hitachi 7500 transmission electron microscope (Hitachi Co., Japan).

### Immunofluorescence Microscopy and Immunohistochemistry

Renal cortex tissues were embedded in Tissue-Tek O.C.T. Compound, snap-frozen in liquid nitrogen and cut in a cryostat (Leica CM 3050S, Germany). The sections were stained with mouse anti-rat nephrin (mAb 5-1-6, 1∶400, a gift from professor Hiroshi Kawachi in Niigata University, Japan) and rabbit anti rat podocin (1∶100), followed by FITC-conjugated goat anti-mouse IgG (1∶50, DAKO). All the sections were examined by immunofluorescence microscopy (Nikon Eclipse E800, Japan).

For immunohistochemical analysis of desmin expression, sections were incubated with mouse monoclonal antibody against rat desmin (D33, DAKO) for 1 h. Then the sections were incubated with HRP-conjugated secondary antibody (Envision kit, DAKO) for 40 min. Color was developed by incubation with AEC (DAKO) and the sections were counterstained with hematoxyline. All the sections were examined by a microscope (Nikon E800, Japan) and all exposure settings were kept constant for each section. For semiquantitative immunohistochemical analysis, 25 glomeruli were selected randomly for each rat and two investigators independently did a blinded immunohistochemical image analysis. The degree of glomerular staining was graded according to the positively stained glomerular area expressed as a percentage of total area: 0, no lesions; 1+, 1–25%; 2+, 25–50%; 3+, 50–75%; 4+, 75–100%. An overall staining score per rat was obtained by multiplying each score (0 to 4+) with the percentage of glomeruli displaying the same degree of positive staining and summing these scores.

### Detection of Renal Cell Apoptosis by TUNEL Staining

For apoptosis assay, 6-µm frozen sections were stained with terminal transferase-mediated dUTP nick end-labeling reagent (Promega, USA) for in situ apoptosis detection. In brief, 6-µm frozen sections were treated with 20 µg/mL proteinase K and then incubated in a nucleotide mixture containing fluorescein-12-dUTP and terminal deoxynucleotidyl transferase. Positive controls were pretreated with 1 U/mL DNAse, and negative controls were incubated without terminal deoxynucleotidyl transferase. Forty high-power fields (400×) were randomly selected. The mean of apoptotic cells per high-power field was counted. Results for apoptotic glomerular cells were expressed as average numbers of TUNEL-positive cells per glomerulus. Results for apoptotic tubular cells were expressed as average numbers of TUNEL-positive cell per high-power field.

### Statistical Analyses

All data are mean±SD of the number of independent measurements. Table 1 is analyzed by an oneway ANOVA. Figures 1 and 3 are analyzed by repeated measures ANOVAs. Figure 6 and figure 7 are analyzed by a 2-way ANOVA and the Bonferroni correction method is used for post-hoc analysis. Values were considered significant if P<0.05.
